# Combined Blockade of Smad3 and JNK Pathways Ameliorates Progressive Fibrosis in Folic Acid Nephropathy

**DOI:** 10.3389/fphar.2019.00880

**Published:** 2019-08-09

**Authors:** Mengjie Jiang, Jinjin Fan, Xinli Qu, Songhui Li, Susan K. Nilsson, Yu Bo Yang Sun, Yaping Chen, Di Yu, Dan Liu, Bi-Cheng Liu, Mingliang Tang, Wei Chen, Yi Ren, David J. Nikolic-Paterson, Xiaoyun Jiang, Jinhua Li, Xueqing Yu

**Affiliations:** ^1^Department of Pediatrics, the First Affiliated Hospital, Sun Yat-sen University, Guangzhou, China; ^2^Department of Anatomy and Developmental Biology, Monash Biomedicine Discovery Institute, Monash University, Clayton, VIC, Australia; ^3^Department of Nephrology, the First Affiliated Hospital, Sun Yat-sen University, Guangzhou, China; ^4^Key Laboratory of Nephrology, National Health Commission and Guangdong Province, Guangzhou, China; ^5^Biomedical Manufacturing Commonwealth Scientific and Industrial Research Organisation (CSIRO), Melbourne, VIC, Australia; ^6^Australian Regenerative Medicine Institute, Monash University, Clayton, VIC, Australia; ^7^Infection and Immunity Program, Monash Biomedicine Discovery Institute and Department of Biochemistry and Molecular Biology, Monash University, Clayton, VIC, Australia; ^8^Institute of Nephrology, Zhong Da Hospital, Southeast University, Nanjing, China; ^9^Key Laboratory for Developmental Genes and Human Disease, Ministry of Education, Institute of Life Sciences, Southeast University, Nanjing, China; ^10^Florida State University College of Medicine, Tallahassee, FL, United States; ^11^Department of Nephrology, Monash Health and Monash University Department of Medicine, Clayton, VIC, Australia; ^12^The Second Clinical College and Shunde Women and Children Hospital, Guangdong Medical University, Shunde, China; ^13^Guangdong Academy of Medical Science, Guangdong Provincial People’s Hospital, Guangzhou, China

**Keywords:** tubulointerstitial fibrosis, Smad3, JNK1/2, PGC-1α, mitochondrial dysfunction

## Abstract

Acute kidney injury leading to chronic kidney disease through tubulointerstitial fibrosis is a major challenge in nephropathy. Several signaling pathways promote interstitial fibrosis; however, effective suppression of fibrosis may require blockade of more than one pathway. This study investigated whether blockade of Smad3 and c-Jun N-terminal kinase (JNK) signaling gives added suppression of interstitial fibrosis in folic acid nephropathy. A single high dose of folic acid (FA) causes acute tubular damage in C57BL/6J mice followed by interstitial fibrosis and chronic renal impairment. Co-activations of Smad3 and JNK signaling occur in both tubular epithelial cells and myofibroblasts in areas of tubulointerstitial damage and fibrosis in both murine FA-induced nephropathy and human IgA nephropathy. Groups of mice were treated with a Smad3 inhibitor (SIS3), a JNK inhibitor (SP600125), or a combination from day 6 after FA administration until being killed on day 28. Each drug efficiently inhibited its specific target (Smad3 phosphorylation or c–Jun phosphorylation) without affecting the other pathway. Given alone, each drug partially reduced renal fibrosis, whereas the combination therapy gave an additive and profound protection from renal fibrosis and improved renal function. Inhibition of Smad3 and/or JNK signaling activities prevented down-regulation of PGC-1α in tubular epithelial cells and up-regulation of PGC-1α in myofibroblasts during FA-induced renal fibrosis and inflammation. The expression of PGC-1α was upregulated in *Smad3* *^−/−^* NRK52E cells while downregulated in *Smad3* *^−/−^*NRK49F cells, suggesting that Smad3 signaling may regulate expression of PGC-1α in renal tubular epithelial cells and fibroblasts in distinct fashion. *In vivo* and cell culture studies also indicate that Smad3 and JNK signaling cooperate to cause mitochondrial dysfunction and cell damage in tubular epithelial cells *via* direct actions on the transcription of PGC-1α. These pathways also act cooperatively to promote renal fibroblast proliferation in tempo-spatial fashion. In conclusion, we have identified a potential combination therapy for progressive renal fibrosis which operates, in part, through modifying mitochondrial function.

## Introduction

There is increasing evidence that acute kidney injury (AKI) is often followed by tubulointerstitial damage due to incomplete renal recovery. Renal tubulointerstitial damage, characterized by tubular atrophy, inflammatory cell infiltration, fibrogenesis, and peritubular capillary rarefaction, is considered the final common pathway leading from chronic kidney disease (CKD) to end-stage renal disease (ESRD) (Takaori and Yanagita, 2016; Kaballo et al., 2017). Many patients develop CKD or even subsequent ESRD due to incomplete recovery after an AKI episode (Takaori and Yanagita, 2016; Kaballo et al., 2017). The progression of CKD to ESRD is a complex process involving various intracellular signaling pathways operating in a tempo-spatial manner. We lack specific anti-fibrotic treatments to stop or retard the progression of CKD to ESRD. A better understanding of the role that signaling pathways play in tubulointerstitial damage will enable rational intervention strategies for CKD.

Inflammation and fibrogenesis are two major determinants in the progression of CKD. The c-Jun N-terminal kinase (JNK) and Smad3 signaling pathways have been implicated in the production of proinflammatory mediators and in the fibrogenesis, respectively. Blockade of JNK signaling reduces the degree of interstitial fibrosis in the obstructed kidney (Ma et al., 2007), and JNK activation in the tubulointerstitium correlates with interstitial fibrosis and loss of renal function in human kidney disease (De Borst et al., 2007). On the other hand, blockade of Smad3 signaling has also been shown to suppress the development of renal interstitial fibrosis (Sato et al., 2003; Li et al., 2010).

Kidney function involves a high metabolic demand in which tubular cells require mitochondrial ATP production to move solutes against electrochemical gradients (Sjostrand and Rhodin, 1953; Weidemann and Krebs, 1969; Tran et al., 2011). Tubular ATP depletion due to mitochondrial dysfunction is a hall mark of AKI and cause of tubular cell injury and death (Thadhani et al., 1996). Disruption of mitochondrial homeostasis and suppression of mitochondrial biogenesis (MB) persists after AKI (Funk and Schnellmann, 2012) and may be a driver of CKD. PPARγ coactivator–1α (PGC-1α) (Puigserver et al., 1998; Wu et al., 1999), the regulator of MB and metabolism, is highly expressed in the kidney. The expression of PGC-1α is markedly suppressed during AKI induced by folic acid (FA), ischemia/reperfusion, or glycerol, while overexpression of PGC-1α in tubular epithelial cells can restore mitochondrial and cellular function and decrease epithelial–mesenchymal transition after oxidative stress (Rasbach and Schnellmann, 2007; Hallman et al., 2008; Yuan et al., 2012), suggesting that PGC-1α may play a pivotal role in the recovery after AKI and in the fibrotic response. Of note, Smad3 deficiency promotes MB and increases basal respiration in adipocytes and Smad3 acts as a repressor of PGC-1α expression. In addition, use of an anti-TGF-β1 neutralizing antibody to block TGF-β/Smad3 signaling protects mice from obesity, diabetes, and hepatic steatosis (Tan et al., 2011; Yadav et al., 2011). We have also demonstrated that Smad3 deficiency decreases high-fat diet–induced mitochondrial injury in podocytes (Sun et al., 2015). Activation of the JNK/c-Jun pathway by potassium deprivation down-regulates PGC-1α expression in cerebellar granule neurons (Liang et al., 2010), and JNK inhibition protects LPS-treated mice from fatty acid oxidation reduction and cardiac dysfunction (Drosatos et al., 2011). Taken together, the above studies indicate that blockade of Smad3 and/or JNK signaling has the potential to reduce mitochondrial dysfunction in AKI. The unanswered question is whether JNK and/or Smad3 signaling pathways modulate the expression of PGC-1α and mitochondrial function during the transition of AKI to CKD.

Here, we report that combined blockade of JNK and Smad3 signaling provides a significant added benefit in suppressing tubular damage and interstitial fibrosis in the AKI to CKD transition in the mouse model of folic acid nephropathy. This protection against ongoing tubular damage was associated with increased PGC-1α expression and improved mitochondrial function *in vivo*, while cell culture studies identified that both pathways modulate PGC-1α promoter activity in tubular epithelial cells. These data identify a novel combination therapy to halt the progression of AKI to CKD and propose a new mechanism of action by which JNK and Smad3 signaling promote renal interstitial fibrosis.

## Methods

### Experimental Animals

C57BL6/J mice of 8 weeks old weighing 20–30 g were purchased from Animal Services, Sun Yat-sen University, Guangzhou, China. Mice were administered FA (Sigma-Aldrich, St. Louis, MO), by intraperitoneal injection (i.p). at the dose of 250-mg/kg body weight dissolved in 0.3 mmol/L sodium bicarbonate. The dose of FA-induced acute tubular necrosis achieved a low mortality rate (about 5%) within 3 days after FA injection. Control mice were administered an equivalent volume of sodium bicarbonate (buffer) by i.p. Body weight of each mouse was measured every day for the whole course of experiment after injection. FA and control mice were sacrificed at 2 days, 6 days, 2 weeks, and 4 weeks after injection, and blood, urine, and kidney tissue were collected at the time of the sacrifice for analysis (*n* = 6/group/time point). In the intervention study, FA mice received JNK-specific inhibitor (SP600125, 15 μg/g/day, Abcam, Cambridge, UK) or/and Smad3-specific inhibitor (SIS3, 5 μg/g/day, Sigma-Aldrich) dissolved in 0.5% carboxymethylcellulose sodium (vehicle) or given vehicle alone by daily i.p. on day 7 after FA injection for 3 weeks. In the co-administration group, the drugs were combined together so that the number and volume of i.p. injections were the same across all groups. Mice were killed at 4 weeks after FA injection (*n* = 6/group). Blood, urine, and kidney samples were collected from each animal. All mice were acclimated in metabolic cages with free access to food and water for collection of 24-h urine samples. Measurement of urine protein and creatinine were determined using a detergent compatible protein assay kit (Bio-Rad, Hercules, CA) and Creatinine Assay Kit (Cayman Chemical, Ann Arbor, MI) according to instructions. Proteinuria was normalized for creatinine excretion. Each kidney was divided into three parts for (Kaballo et al., 2017) immunoprecipitation/Western blotting; (Takaori and Yanagita, 2016) 10% buffered formalin-fixed, paraffin-embedded tissue; and (Ma et al., 2007) periodate-lysine-paraformaldehyde, OCT-embedded tissue. All experiments were performed at Animal Services, Sun Yat-sen University and approved by the Sun Yat-sen University Animal Ethics Committee.

### Human Renal Biopsy Specimens

Studies using human tissue were approved by the Human Ethics Committee of Monash Medical Centre and the Human Ethics Committee of the 1^st^ Affiliated Hospital, Sun Yat-sen University. Human ethics committee approval and written informed consent from the patients were obtained in both institutions. Cryostat sections of snap-frozen tissue, excess to that required for diagnosis, were examined in five cases of IgA nephropathy. Normal kidney tissue was obtained from the non-involved pole of nephrectomies performed as the result of renal carcinoma.

### Histology and Confocal Microscopy

Kidneys were fixed in 10% buffered formalin, and paraffin-embedded tissue sections (4 μm) stained with Masson trichrome. Cryostat sections of tissues (4 μm) fixed in periodate-lysine paraformaldehyde were blocked with 2% BSA in PBS and incubated with the following antibodies: rabbit anti-fibronectin (Sigma) or rabbit anti-collagen IV (Southern Biotechnology, Birmingham, AL) followed by goat anti-rabbit Alexa Fluor 488 (Invitrogen, Mount Waverly, Australia) and Cy3-conjugated mouse anti-α-SMA antibody (Sigma) or Alexa Fluor 488-conjugated Rabbit Anti-Tomm20 antibody (Abcam); rat anti-Smad3 (R&D Systems, Minneapolis, MN) followed by goat anti-rat Alexa Fluor 488, rabbit anti-phosphorylated JNK1/2 (Cell Signaling Technology, Danvers, MA) followed by goat anti-rabbit Alexa Fluor 647, and Cy3-conjugated mouse anti-α-SMA antibody; or rat anti-CD11b (Abcam) followed by goat anti-rat Alexa Fluor 488. Sections were counterstained with 4,6-diamidino-2-phenylindole (DAPI, Sigma) to visualize nuclei and analyzed with an Olympus FluoView 1000 Confocal Microscope (Olympus, Tokyo, Japan) under an oil UPLFL × 60 or × 40 objective (NA 1.25; Olympus) using the FV10-ASW software (V1.3c; Olympus). The percentage of staining area was counted in blinded slides. The number of infiltrating interstitial CD11b(+)/DAPI(+) cells was quantified in 20 non-overlapping cortical fields/each kidney and expressed as cells per mm^2^ of cortical interstitium. Images were analyzed by ImageJ (http://rsb.info.nih.gov/ij/). 

### Cell Culture

Wild-type C57BL6/J (WT) mouse renal fibroblasts, NRK52E cells, and NRK49F cells were cultured as previously described (Li et al., 2010; Qu et al., 2012).

### Western Blot Analysis

Kidneys (∼20 mg) were homogenized and suspended in 0.4 ml of lysis buffer containing 10 mmol/L Tris-HCL, pH 7.4, 1% Triton X-100, 0.5% deoxycholate, 1 mmol/L phenylmethyl sulfonyl fluoride, and cOmplete™, EDTA-free Protease Inhibitor Cocktail (Roche, Castle Hill, Australia). Cultured cells (∼2 × 10^6^) were lysed in 0.3 ml RIPA lysis buffer and sonicated. Protein concentration estimations were performed with a detergentcompatible protein assay kit (Bio-Rad, Hercules, CA). Proteins (∼50 µg) were separated by 10% sodium dodecyl sulfate-polyacrylamide gel electrophoresis and transferred to a polyvinylidene difluoride membrane. After blocking for 30 min at 4°C in 5% bovine serum albumin in phosphate-buffered saline with 0.1% Tween 20, the membrane was incubated overnight with rabbit anti-JNK, p-JNK (Cell Signaling Technology), or rabbit anti-Smad3, p-Smad3 (Biorbyt, Cambridge, UK). Blots were then incubated with peroxidase-conjugated goat anti-rabbit IgG for 1 h at room temperature, and bound antibody was detected using an ECL Kit (Amersham Pharmacia Biotech, Arlington, IL) and the Kodak 4000MM Image Station. Band density was quantified using ImageJ (http://rsb.info.nih.gov/ij/). 

### Enzyme-Linked Immunosorbent Assay (ELISA)

Mouse Cystatin C DuoSet ELISA Kit (R&D Systems) was used to measure the protein level of cystatin c in serum according to the instruction supplied.

### Collagen I/PGC-1α Promoter Luciferase Assay

1 day after collagen I promoter luciferase plasmid or PGC-1α promoter luciferase plasmid was transfected NRK52E cells or NRK49F cells by lipofectamine 2000. Cells were treated with or without TNF-α, TGF-β1, DMSO, Sp600125, and/or SIS3 for 18 h. Wild-type *Renilla luciferase* was used for normalization in Reporter Assays. Then, cells were harvested and luciferase assay was performed according to the instruction in Luciferase Assay Kit (Promega).

### Isolation of Mitochondria From Tissues and Cells

We isolated mitochondria using the *Mitochondria Isolation Kit* for fresh tissue (Thermo Scientific, cat. no. 89801) and cells (Thermo Scientific, cat. no. 89874).

### Cell Proliferation Assay

Cell proliferation assay was performed using Cell Proliferation ELISA, BrdU (colorimetric) Kit (Roche Applied Science, Indianapolis, IN). Briefly, the cells were cultured in 96-well plates at a density of 6,000 cells/100 μl/well in complete growth media. The sense sequence of rat PGC-1α siRNA was: 5’AAGACGGATTGCCCTCATTTG. The negative control (scramble) siRNA sequence was: 5’AAGCTTCATAAGGCGCATAGC. The SiRNA was transfected into NRK52E cells or NRK49F cells by using lipofectamine transfection reagent according to manufacturer’s instructions. After 24 h, NRK52E cells and NRK49F cells were treated with epidermal growth factor (EGF) (50 ng/ml; R&D Systems, MN, USA) or PDGF-BB (20 ng/ml; R&D Systems), respectively. After 48 h, the cells were labeled using 10 μM BrdU per well and cultured for another 24 h. The detection and measurement of cell proliferation were performed according to the instruction in Cell Proliferation ELISA Kit.

### Lentiviral CRISPR/Cas9 Constructs

For the inducible Smad3 sgRNA constructs, the previously described FgH1tUTG plasmid was modified to contain the rat Smad3 sgRNA (5’TCCCTACAAGGCGGCACATTGGGA3’ and 5’ AAACTCCCAATGTGCCGCCTTGTA’) cassette, which was inserted into bi-directional Bsmb1 sites linked to the GFP fluorescent protein (Aubrey et al., 2015). The constitutive Cas9 expression vectors were derived from the pFUGW, Cas9 protein linked *via* the T2A peptide to the mCherry fluorescent reporter protein (Aubrey et al., 2015).

### Statistical Analysis

Data are expressed as mean ± SD with statistical analyses performed using two-way analysis of variance or one-way analysis of variance, with *post hoc* analysis with Tukey’s multiple comparison test using GraphPad Prism 6.0 (Graph-Pad Software, San Diego, CA). A probability (P) value below 0.05 was accepted as statistically significant.

## Results

Development and progression of tubulointerstitial fibrosis and inflammation following FA-induced acute kidney failure

**Figure 1:**
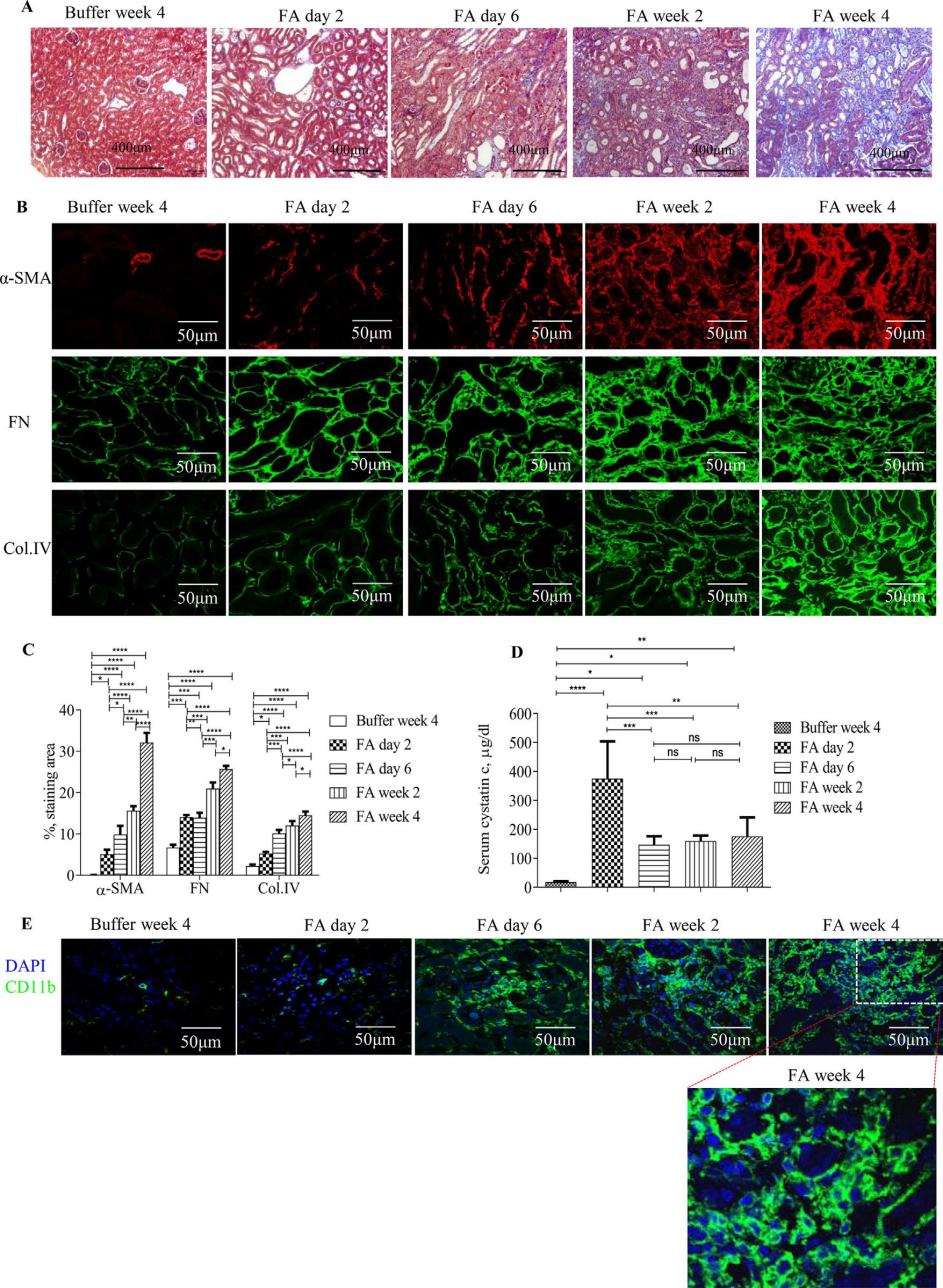
Development and progression of tubulointerstitial fibrosis and inflammation following folic acid (FA)–induced acute kidney failure. **(A)** Masson trichrome staining showing histological changes in the kidneys 2 days, 6 days, 2 weeks, and 4 weeks after folic acid or buffer injection. **(B)** Confocal microscopy showing expression of α-SMA, fibronectin (FN), and collagen IV (Col. IV) and **(E)** CD11b in the kidneys 2 days, 6 days, 2 weeks, and 4 weeks after folic acid or buffer injection. **(C)** Quantification of α-SMA, FN, and Col. IV staining area in the kidneys 2 days, 6 days, 2 weeks, and 4 weeks after folic acid or buffer injection. **(D)** Time course of serum cystatin c changes from mice after folic acid or buffer injection. **(E)** Confocal microscopy showing expression of CD11b after folic acid or buffer injection. Original magnification; ×400 **(A **and **E)**; ×600 **(B)**. Data are mean ± SD (*n* = 6/group). *P < 0.05, **P < 0.01, ***P < 0.001, ****P < 0.0001.

Masson trichrome staining and immunostaining showed that administration of FA to mice resulted in severe tubulointerstitial injury, development, and progression of tubulointerstitial fibrosis and inflammation (**Figures 1A–C, E**). Levels of serum cystatin c dramatically increased by 2 days after FA administration and then dropped but remained at a higher level than that of vehicle administration throughout the study period (**Figure 1D**), demonstrating acute renal failure followed by incomplete tubulointerstitial recovery and the development and progression of CKD.

### Smad3 and JNK Signaling Co-activation in FA-Induced Nephropathy and in Tubulointerstitial Fibrosis in Human IgA Nephropathy

**Figure 2:**
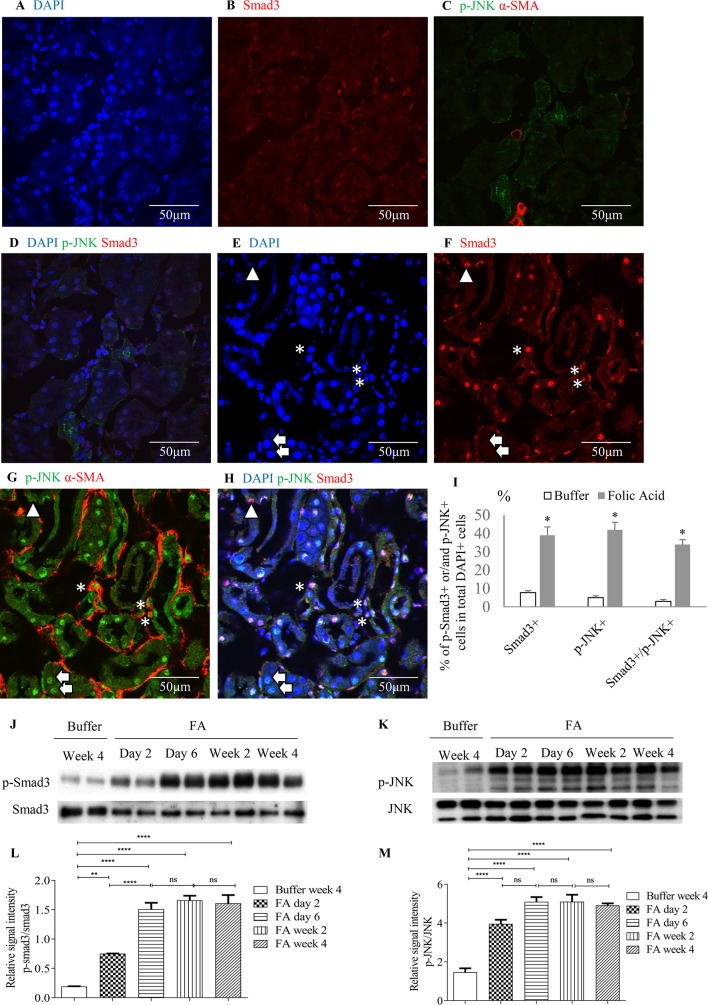
Smad3 and JNK signaling activation after folic acid (FA)–induced acute kidney injury. Confocal microscopy demonstrated same fields in buffer-treated kidney **(A**–**D)** or kidney 6 days after folic acid treatment **(E**–**H)**. **(A)** DAPI, blue. **(B)** Nuclear staining of Smad3, red. **(C)** p-JNK, green and α-SMA, red. **(D)** Merged with DAPI (blue), nuclear staining of Smad3 (red) and p-JNK (green). **(E)** DAPI, blue. **(F)** Nuclear staining of Smad3, red. **(G)** p-c-Jun N-terminal kinases (JNK), green and α-SMA, red. **(H)** Merged with DAPI (blue), nuclear staining of Smad3 (red) and p-JNK (green). Arrows indicate nuclear Smad3(−)/p-JNK(+) cells; arrow head shows a nuclear Smad3(+)/p-JNK(−) cell; asterisks show interstitial α-SMA(+)/nuclear Smad3(+)/p-JNK(+) cells. **(I)** Quantification of nuclear staining of nuclear Smad3(+), p-JNK(+), and nuclear Smad3(+)/p-JNK(+) cells. *P < 0.05 *vs.* buffer-treated kidney. Western blotting shows p-Smad3 **(J)** and p-JNK **(K)** in kidneys 2 days, 6 days, 2 weeks, and 4 weeks after folic acid or buffer injection. Quantification of relative signal intensities of p-Smad3/Smad3 **(L)** and p-JNK/JNK **(M)** in kidneys after folic acid or buffer injection. Original magnification (×600). Data are mean ± SD (*n* = 6/group). **P < 0.01; ****P < 0.0001; ns, not significant.

**Figure 3:**
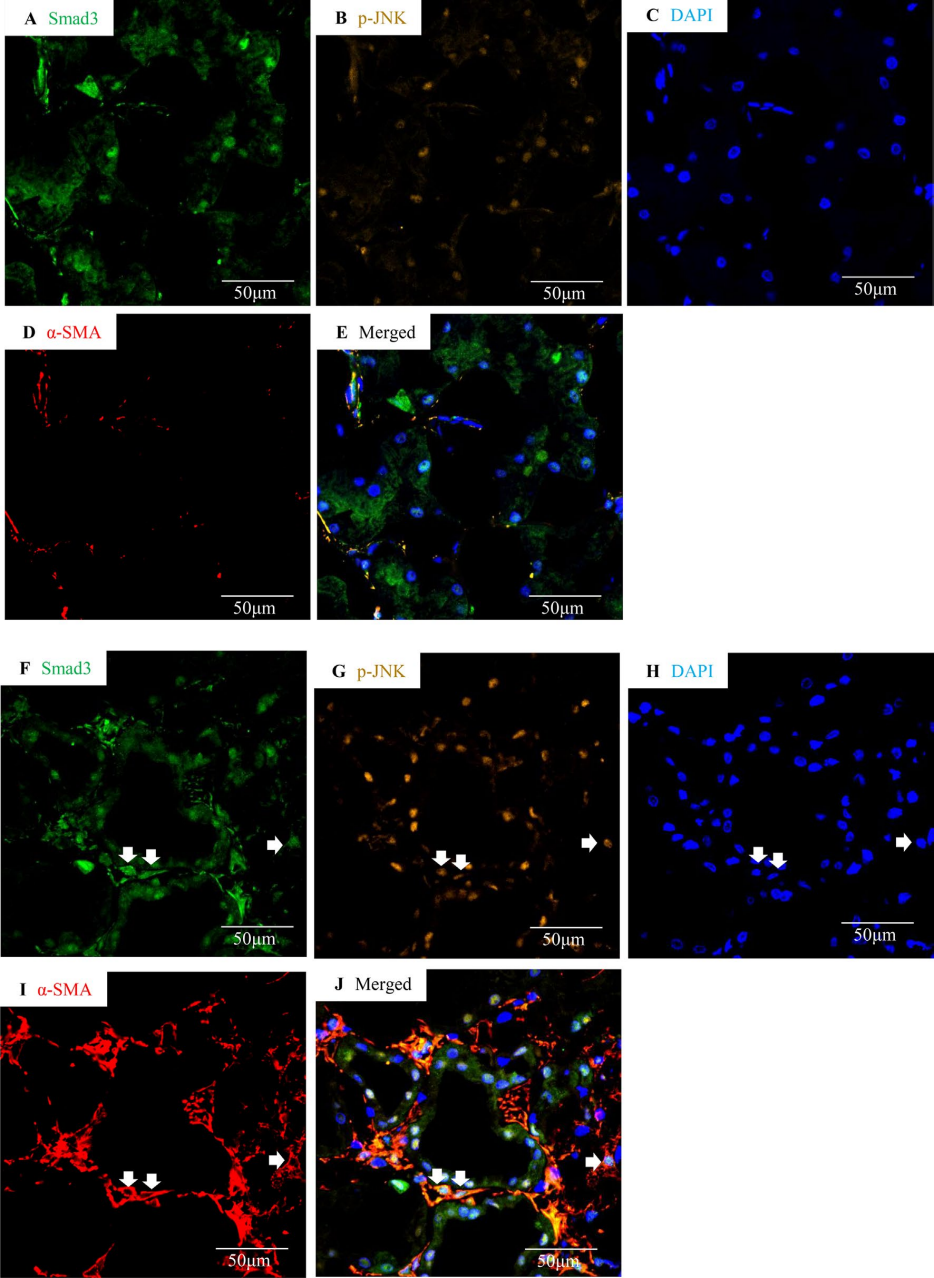
Smad3 and JNK signaling co-activation in human IgA nephropathy. Multi-color confocal microscopy of the cortical interstitium in normal human kidney **(A**–**E)** and an area of tubulointerstitial fibrosis in a case of IgA nephropathy **(F**–**J)**. **(A **and **F)** nuclear staining of Smad3, green; **(B **and **G)** p-JNK, yellow; **(C **and **H)** DAPI, blue; **(D **and **I)** α-smooth muscle actin, α-SMA, red; **(E **and **J)** merged with nuclear Smad3 (green), p-JNK (yellow), DAPI (blue), and α-SMA (red). Arrows indicate interstitial α-SMA(+)/nuclear Smad3(+)/p-JNK(+) cells. Original magnification (x600).

Confocal microscopy demonstrated co-localization of p-Smad3 and p-JNK in tubulointerstitial cells, including tubular epithelial cells and α-smooth muscle actin (α-SMA)-positive myofibroblasts in FA-treated but not in buffer-treated mouse kidney (**Figures 2A–I**). Western blotting further demonstrated that phosphorylated Smad3 (p-Smad3) and JNK (p-JNK) levels were significantly upregulated 2 days after administration of FA, and this upregulation persisted during the progression of renal inflammation and fibrosis (**Figures 2J–M**). Confocal microscopy also demonstrated that few p-Smad3(+) and p-JNK(+) cells were seen in normal human kidney from the uninvolved pole of carcinoma nephrectomy samples (**Figures 3A–E**). However, numerous tubular cells and α-SMA(+) myofibroblasts exhibited p-Smad3(+) or/and p-JNK(+) staining in biopsy specimens of IgA nephropathy (**Figures 3F–J**), suggesting that co-activation of Smad3 and JNK1/2 signaling in the tubulointerstitium persisted during the development and progression of CKD.

### Inhibition of Smad3 and/or JNK Signaling Activities Protected Mice From FA-Induced Development and Progression of Renal Fibrosis and Inflammation

**Figure 4:**
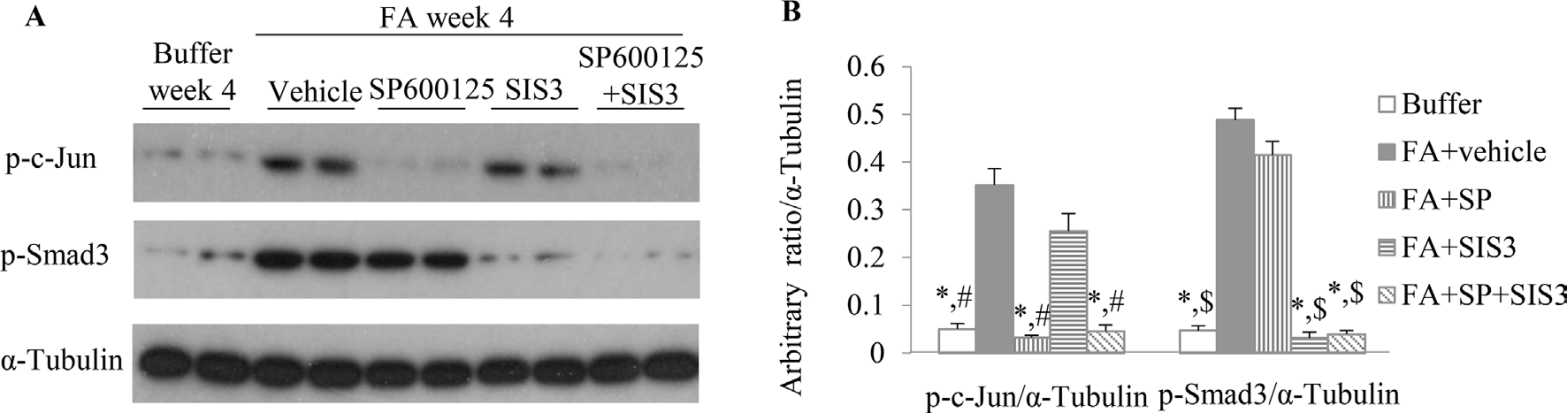
The impact of blockade of JNK and/or Smad3 signaling on folic acid (FA)–induced kidney injury. **(A** and **B)** Western blotting shows p-c-Jun, p-Smad3, and α-tubulin in kidneys from buffer-treated kidneys and FA-induced kidney disease treated with vehicle, SP600125, SIS3, or SP600125 + SIS3. **(B)** Quantification of Western blot results. *P < 0.05 vs. FA+vehicle-treated group;^#^ *P < 0.05 vs.* FA+SIS3-treated group; *^$^* *P < 0.05 vs.* FA+SP-treated group.

To investigate the functional role of Smad3 and/or JNK signaling pathways in the pathogenesis of FA-induced renal tubulointerstitial fibrosis and inflammation, a Smad3-specific inhibitor (SIS3) and/or a JNK inhibitor (SP600125) were administered daily to mice, beginning on day 6 days after FA injection. Western blotting demonstrated that SIS3 and SP600125 abrogated phosphorylation of Smad3 and c-Jun, respectively (**Figures 4A, B**), indicating that a maximally effective drug dose was used in each case. However, SIS3 did not affect c-Jun phosphorylation, and SP600125 did not affect Smad3 phosphorylation (**Figures 4A, B**), indicating that these signaling pathways operate in a largely independent fashion.

**Figure 5:**
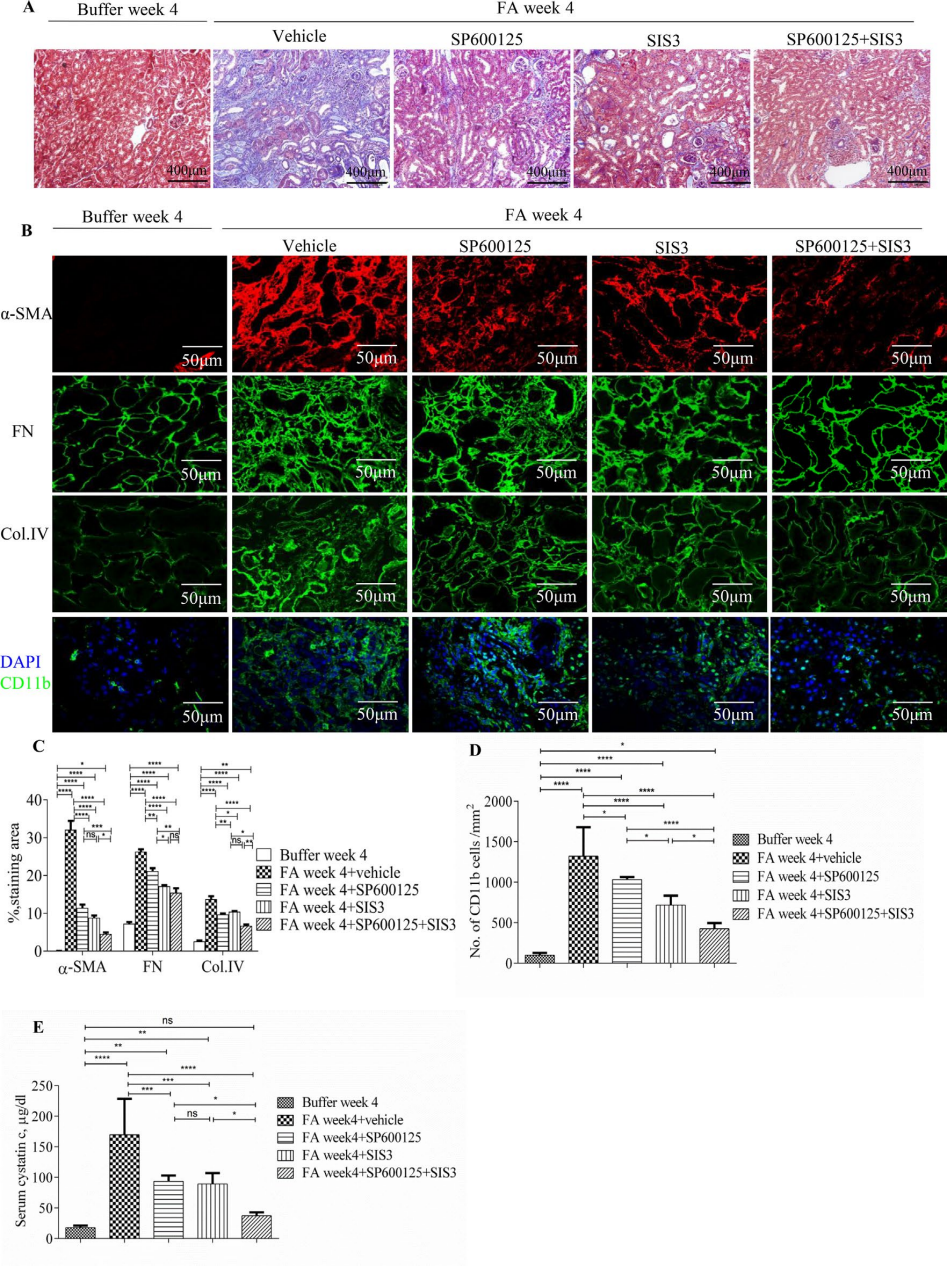
Inhibition of Smad3 and/or JNK signaling reduces renal fibrosis and inflammation in folic acid (FA)–induced kidney injury. **(A)** Masson trichrome staining shows histological changes in the kidneys 4 weeks after buffer or folic acid injection with SP600125, SIS3, or combined SP600125+SIS3 treatment. **(B)** Confocal microscopy shows staining of α-SMA, fibronectin (FN), Col IV, and CD11b in the kidneys 4 weeks after buffer or folic acid injection with different treatments. Magnification: ×400 **(A)**, ×600 **(B** and **E)**. **(C)** Quantification of α-SMA, FN, and Col IV interstitial staining area. **(D)** The number of CD11b(+) macrophages in the kidneys 4 weeks after buffer or folic acid injection with different treatments. **(E)** Serum cystatin c levels in mice 4 weeks after buffer or folic acid injection with different treatments. Data are mean ± SD, (*n* = 6/group). *P < 0.05, **P < 0.01, ***P < 0.001, ****P < 0.0001.

**Figure 6:**
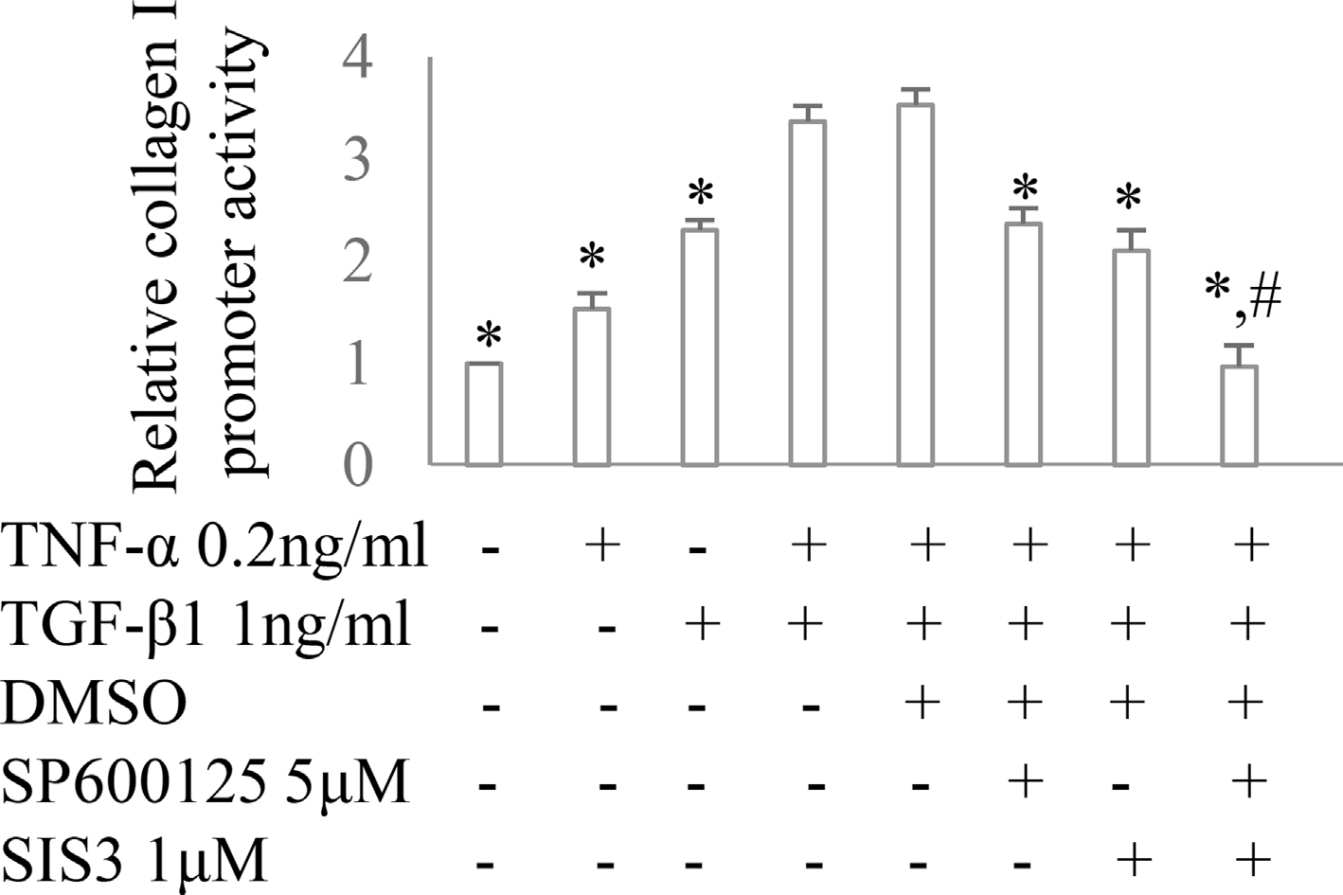
The impact of Smad3 and JNK signaling on TNF-α and/or TGF-β1-induced fibrotic response. Collagen I promoter luciferase activity assay in cultured renal fibroblasts. *P < 0.05 *vs.* TNF-α 0.2 ng/ml + TGF-β1 1 ng/ml or TNF-α 0.2 ng/ml + TGF-β1 1 ng/ml +DMSO; ^#^ *P* < 0.05 *vs.* TNF-α 0.2 ng/ml + TGF-β1 1 ng/ml + SP600125 or TNF-α 0.2 ng/ml + TGF-β1 1 ng/ml + SIS3. Data are mean ± SD. Experiments were repeated three times.

Co-administration of SIS3 and SP600125 inhibited both Smad3 and JNK signaling activations (**Figures 4A, B**). Masson trichrome and confocal microscopy demonstrated that administration of SIS3 alone or SP600125 alone partially reduced tubulointerstitial fibrosis and macrophage infiltration. Co-administration of SIS3 and SP600125 further protected mice from fibrosis and inflammation. Serum cystatin c levels also confirmed the salutary effects of SIS3 and/or SP600125 on renal function (**Figures 5A–E**). Collagen I promoter luciferase assay further demonstrated that TNF-α has additive effect on TGF-β1-induced fibrotic response while blockade Smad3 and/or JNK signaling pathway almost abrogated this fibrotic response in renal fibroblasts (**Figure 6**). Taken together, all these data suggested that Smad3 and JNK signaling pathways may play an essential role in the pathogenesis of tubulointerstitial fibrosis and inflammation and that inhibition of Smad3 and JNK signaling may retard the development and progression of CKD.

### Inhibition of Smad3 and/or JNK Signaling Activities Reduced Mitochondrial Dysfunction and Promoted Renal Tubular Epithelial Cell Recovery From Folic Acid-Induced Kidney Damage

**Figure 7:**
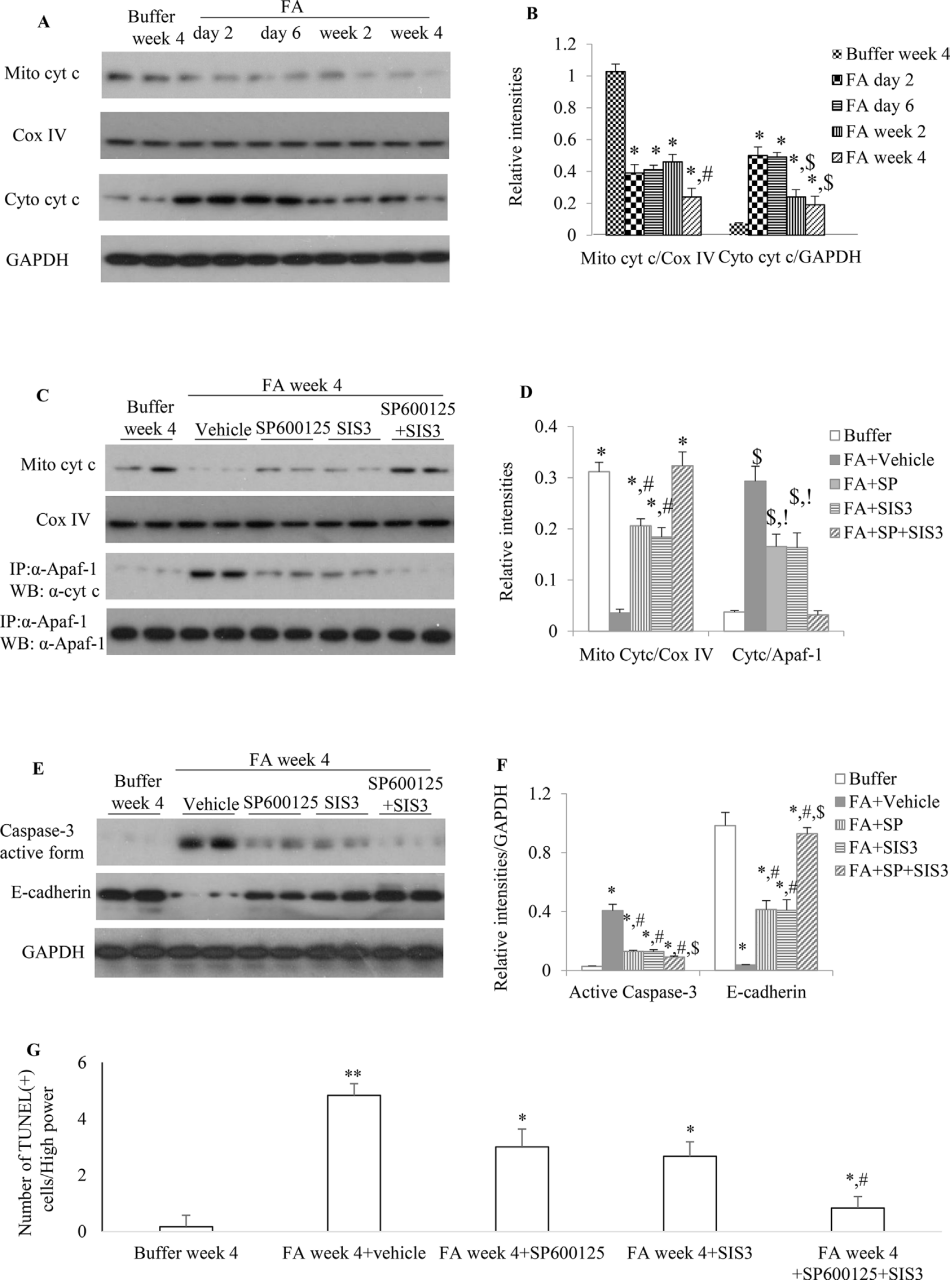
The impact of blockade of JNK and/or Smad3 signaling on folic acid (FA)–induced kidney injury. **(A** and **B)** Western blotting shows mitochondrial cytochrome c and cytosol cytochrome c in buffer-treated or a time-course of FA-treated kidneys. **(B)** Quantification of Western blot results. *P < 0.05 *vs.* buffer week 4. ^#^ *P* < 0.05 *vs.* FA day 2, FA day 6, or FA week 2; *^$^* *P* < 0.05 *vs.* FA day 2 or FA day 6. **(C** and **D)** Western blotting (WB, upper two panels) and immunoprecipitation/WB (IP/WB, lower two panels) show the effect of treatment with SP600125 alone, SIS3 alone, and SP600125 + SIS3 on mitochondrial cytochrome c and interaction of cytochrome c with Apaf-1 in 4-week FA-induced kidney injury. **(D)** Quantification of blots. *P < 0.05 vs. FA+vehicle; *^#^* *P < 0.05 vs. FA+SP+SIS3;* *^$^* *P < 0.05 vs. Buffer week4; !P < 0.05 vs. FA+SP+SIS3.* **(E **and **F)** West blotting shows effect of treatment with SP600125 alone, SIS3 alone, and SP600125 + SIS3 on caspase-3 activation and E-cadherin in 4-week FA-induced kidney injury. **(F)** Quantification of blots. *P < 0.05 *vs. *buffer week 4. ^#^ *P* < 0.05 *vs.* FA + vehicle; *^$^* *P* < 0.05 *vs.* FA+SP600125 or FA + SIS3. Data are mean ± SD. **(G)** Quantitation of TUNEL(+) cells per high power field (100×). *P < 0.05 *vs.* FA week 4; **P < 0.01 *vs.* buffer week 4; ^#^ *P* < 0.05 *vs.* FA week 4 + SP600125 or FA week 4 + SIS3. Data are mean ± SD (*n* = 6/group).

Administration of FA to mice resulted in a rapid reduction of mitochondrial cytochrome c (**Figures 7A, B**) during the early phase of injury and this down-regulation of mitochondrial cytochrome c further reduced 2 weeks after FA administration. The increase protein expression of cytosol cytochrome c, a mitochondrial injury indicator, was demonstrated by WB in which a 3.5-, 3.5-, 1.5-, and 1-fold increase was observed in the kidneys of FA-injected mice at 2, 6, 14, and 28 days, respectively, compared with buffer-treated mice. This dynamic changes of mitochondrial activity demonstrated mitochondrial damage persisted and mitochondria incompletely recovered after FA-induced kidney injury.

Administration of SP600125 alone or SIS3 alone prevented cytochrome c release from mitochondria and decreased the interaction between Apaf-1 and cytosol cytochrome c and the protein expression level of active caspase-3 when compared to vehicle-treated group (**Figures 7C, D**), suggesting that both SP600125 and SIS3 are reducing mitochondrial dysfunction and resultant cell damage, including cell death through apoptosis. Co-administration of SP600125 and SIS3 almost recovered the expression of mitochondrial cytochrome c, significantly decreased the complex of Apaf-1 and cytosol cytochrome c and the formation of active caspase-3 and TUNEL(+) cells (**Figures 7C–G**). Furthermore, administration of SP600125 alone or SIS3 alone decreased the loss of E-cadherin, the tubular epithelial marker (**Figures 7E, F**). Co-administration of SP600125 and SIS3 almost prevented the loss of E-cadherin, an indicator of tubular cell damage, when compared to buffer-treated group (**Figures 7E, F**). Taken together, these studies demonstrated the relationship between Smad3 and JNK signaling pathways, mitochondrial function, and tubular epithelial recovery.

### Inhibition of Smad3 and/or JNK Signaling Activities Prevented Down-Regulation of PGC-1α in Tubular Epithelial Cells and Up-Regulation of PGC-1α in Myofibroblasts During FA-induced Renal Fibrosis and Inflammation

**Figure 8:**
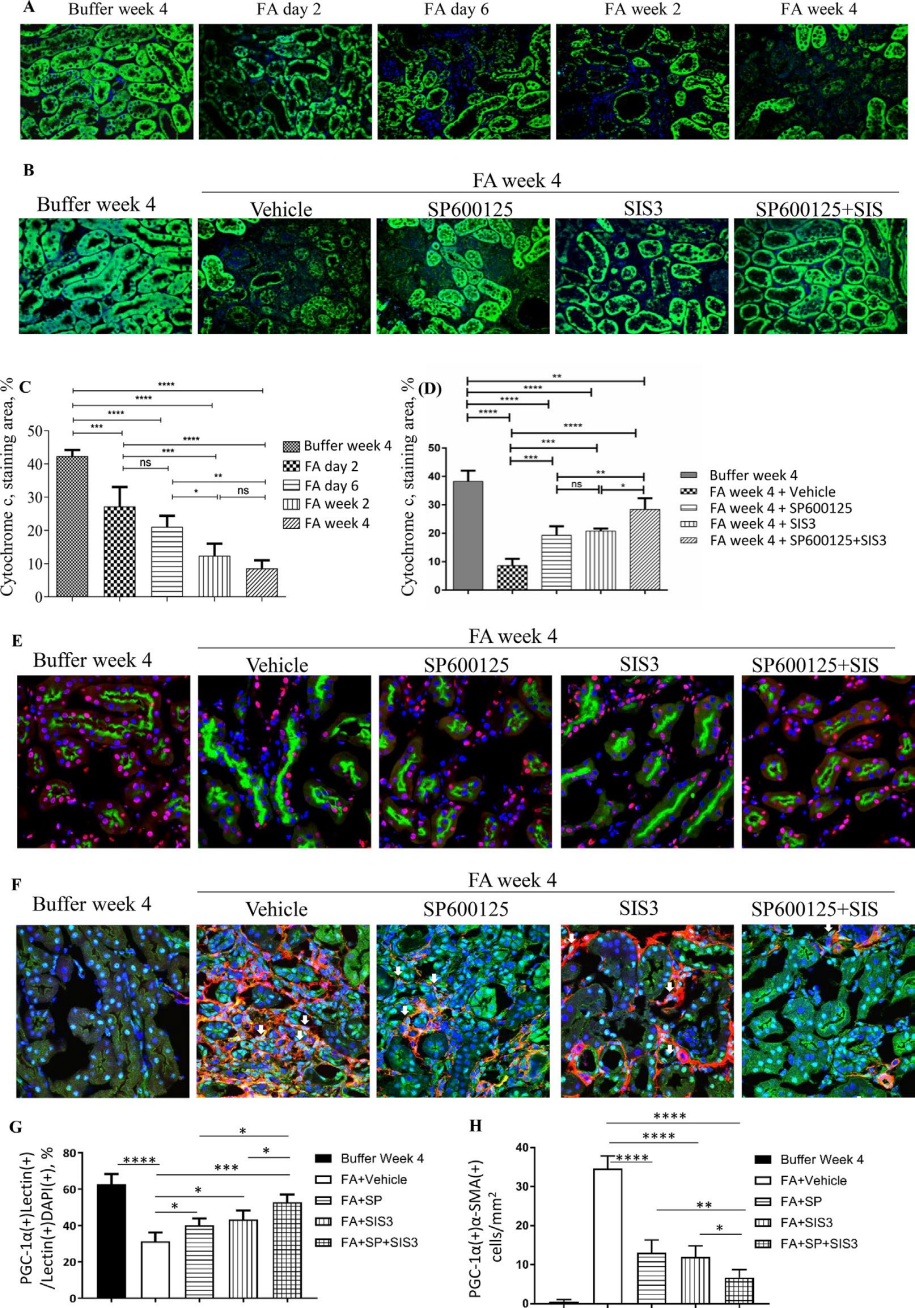
Inhibition of Smad3 and/or JNK signaling reduces mitochondrial dysfunction in folic acid (FA)–induced kidney injury. Confocal microscopy shows cytochrome c (green) and nuclear staining DAPI (blue) in buffer-treated or FA-treated kidneys **(A)** and in kidneys 4 weeks after buffer or FA injection and treated with vehicle, SP600125 (SP), SIS3, or SP+SIS3 **(B)**. Quantification of staining area of cytochrome c **(C** and **D)**. Data are mean ± SD (*n* = 6/group), *P < 0.05; **P < 0.01; ***P < 0.001; ****P < 0.0001. **(E)** Confocal microscopy showing staining of lectin in tubular epithelial cells (green), PGC-1α (red), and nuclear staining DAPI (blue) in kidneys 4 weeks after buffer or folic acid injection and treated with vehicle, SP600125 (SP), SIS3, or SP+SIS3. **(F)** Confocal microscopy showing staining of PGC-1α (green) in myofibroblasts (red), and nuclear staining DAPI (blue) in kidneys 4 weeks after buffer or folic acid injection and treated with vehicle, SP600125 (SP), SIS3, or SP+SIS3. **(G)** Quantification of percentages of PGC-1α(+)lectin(+) cells in total lectin(+)DAPI(+) cells. **(H)** Quantification of numbers of PGC-1α(+)α-smooth muscle actin (α-SMA)(+) cells/mm^2^. Data are mean ± SD (*n* = 6/group). *P < 0.05; **P < 0.01; ***P < 0.001; ****P < 0.0001.

To further investigate the impact of Smad3 and JNK signaling activation on mitochondrial dysfunction, the expression of cytochrome c, the mitochondrial marker, and PGC-1α, the MB marker, were examined. Confocal microscopy demonstrated that there was a rapid reduction of cytochrome c in tubular epithelial cells 2 days after FA injection, and the decrease persisted (**Figures 8A, C**) until the experimental endpoint. Confocal microscopy also demonstrated that the administration of SP600125 or SIS3 reduced loss of cytochrome c and PGC-1α in tubular epithelial cells and decreased the expression of PGC-1α in myofibroblasts (**Figures 8B, D–H**), suggesting co-administration of SP600125 and SIS3 further enhanced the impact on PGC-1α expression.

### Smad3 and JNK1/2 Signaling Pathways Regulated Expression of PGC-1α in Renal Tubular Epithelial Cells and Fibroblasts in Distinct Fashion

**Figure 9:**
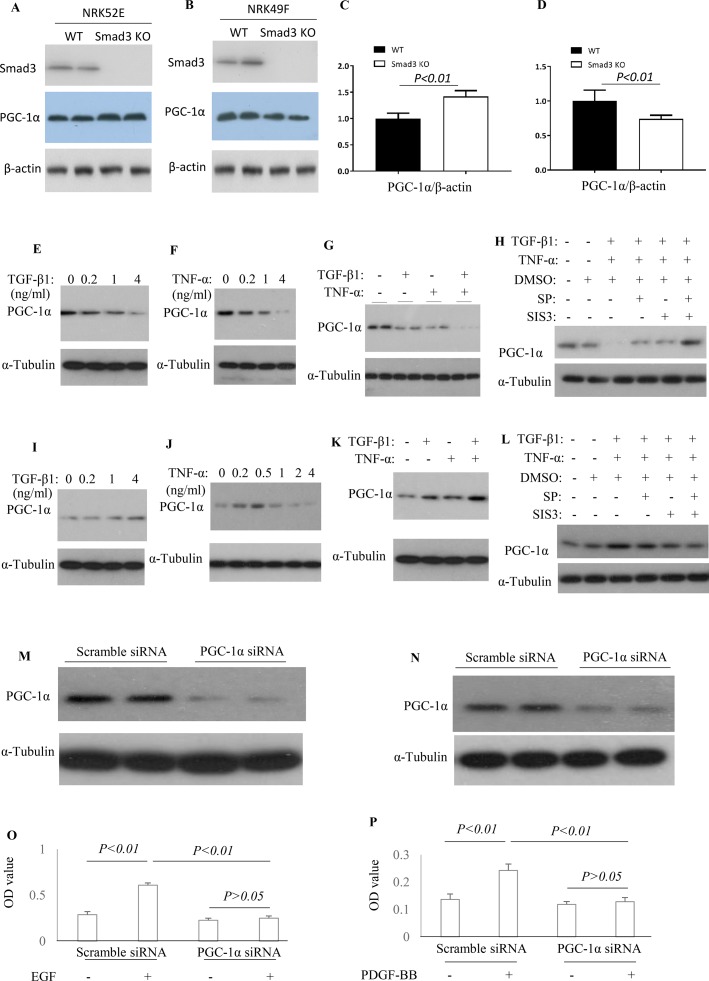
Differential effect of JNK and Smad3 inhibition on PGC-1α expression and proliferation in renal tubular epithelial cells *versus* fibroblasts. Western blotting demonstrated expression of Smad3, PGC-1α, and β-actin in wild type (WT) and *Smad3* *^−/−^* NRK52E cells **(A)** and NRK49F cells **(B)**. Quantification of PGC-1α/β-actin in NRK52E cells **(C)** and NRK49F cells **(D)**. Western blotting identifies PGC-1α expression levels in NRK52E tubular epithelial cells **(E**–**H)** and NRK49F fibroblasts **(I**–**L)** after TGF-β1 and/or TNF-α treatment, with or without addition of SP600125 (SP) and/or SIS3. Experiments were repeated three times. Western blotting demonstrates a marked knock-down of PGC-1α by siRNA treatment in both NRK52E tubular epithelial cells **(M)**, and NRK49F fibroblasts **(N)**. Quantification of EGF-induced proliferation of NRK52E cells **(O)** and of PDGF-BB induced proliferation of NRK49F cells **(P)**, following scramble or PGC-1α siRNA treatment. Data are mean ± SD. Experiments were repeated three times.

To knockout Smad3 in NRK52E cells and NRK49F cells, the inducible Smad3 sgRNA was constructed in Lentiviral CRISPR/Cas9 system. Western blotting demonstrated that Smad3 was knockout in NRK52E cells and NRK49F cells (**Figures 9A, B**). The expression of PGC-1α was upregulated in *Smad3* *^−/−^* NRK52E cells while downregulated in *Smad3* *^−/−^* NRK49F cells (**Figures 9A–D**), suggesting that Smad3 signaling may regulate expression of PGC-1α in renal tubular epithelial cells and fibroblasts in distinct fashion. TGF-β1 or TNF-α decreased the expression of PGC-1α in rat tubular epithelial cells (NRK52E) while TGF-β1 + TNF-α further enhanced the inhibitive effects on NRK52E cells (**Figures 9E–G**). Administration of SP600125 or/and SIS3 prevented TGF-β1-induced or TNF-α-induced down-regulation of PGC-1α in NRK52E cells (**Figure 9H**). TGF-β is known to cause damage to tubular cells. The cell culture findings are consistent with the confocal staining results in the kidney in which there is down-regulation of PGC-1α in tubular cells. TGF-β1 increased the expression of PGC-1α in rat renal fibroblasts (NRK49F). Low dose of TNF-α increased while higher dose TNF-α inhibited the expression of PGC-1α in NRK49F cells. Administration of SP600125 or/and SIS3 prevented the impacts of TGF-β1 and TNF-α on the expression of PGC-1α in NRK49F cells (**Figures 9I–L**). To investigate the relationship between PGC-1α expression and cell proliferation, the expression of PGC-1α was knocked down by PGC-1α siRNA. Knockdown of PGC-1α decreased the EGF-induced proliferation in NRK52E cells (**Figures 9M, O**) and PDGF-BB-induced proliferation in NRK49F cells (**Figures 9N, P**).

### Smad3 and JNK Signaling Pathways Modulated PGC-1α Promoter Activity in Renal Tubular Epithelial Cells and Fibroblasts.

**Figure 10:**
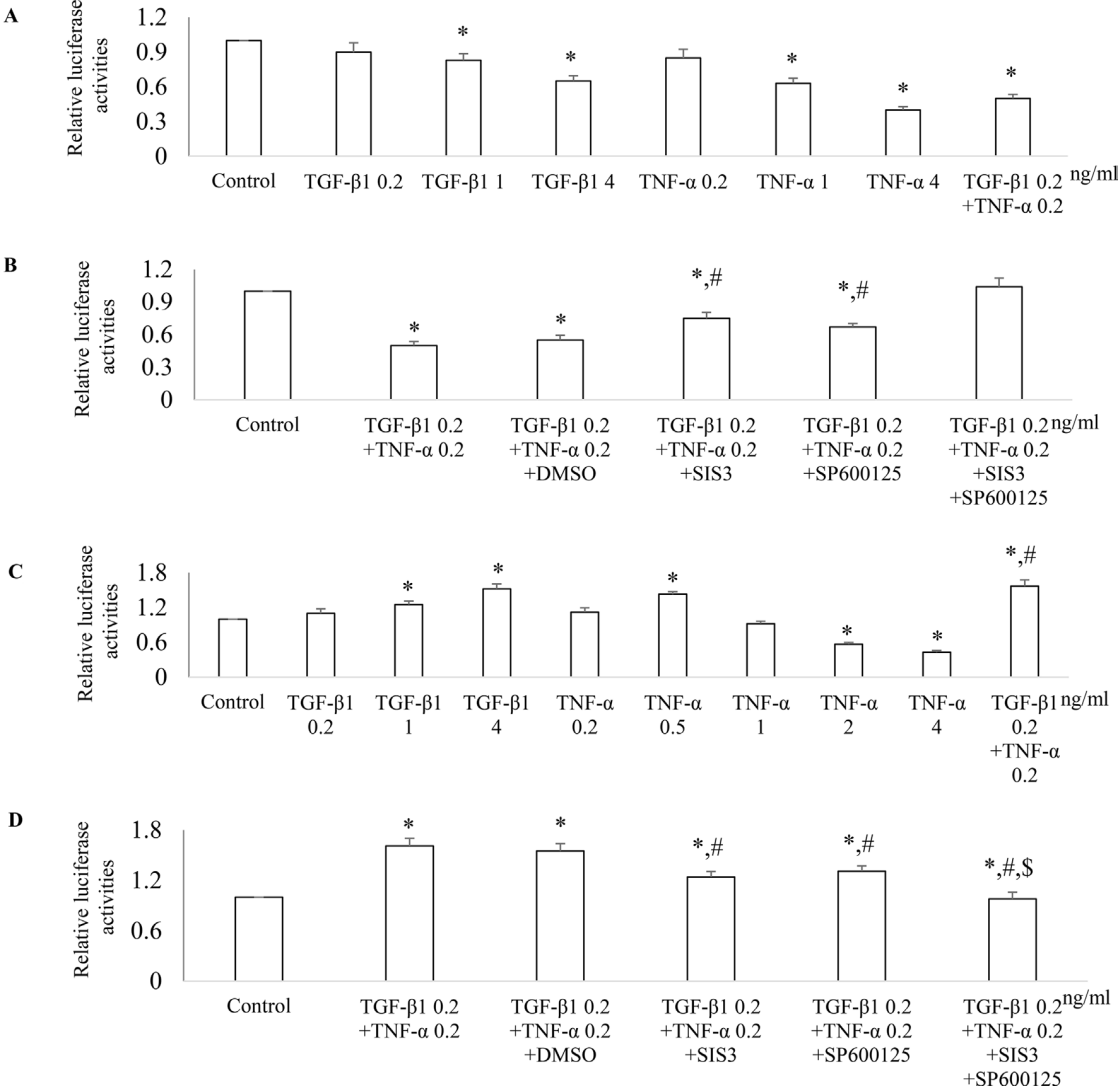
Differential effect of JNK and Smad3 inhibition on PGC-1α promoter activity in renal tubular epithelial cells *versus* fibroblasts. NRK52E tubular epithelial cells **(A** and **B)** and NRK49F fibroblasts **(C** and **D)** were transfected with a PGC-1α promoter luciferase plasmid. 24 h after transfection, NRK52E cells **(A)** and NRK49F cells **(C)** were treated with TGF-β1 and/or TNF-α for 18 h then luciferase activity assay was performed. *P < 0.05 *vs.* control; *^#^* *P* < 0.05 *vs.* TGF-β1 0.2 ng/ ml. 24 h after transfection, NRK52E cells **(B)** and NRK49F cells **(D)** were incubated with vehicle (DMSO), SIS3 1 µM, or/and SP600125 10 µM for 30 min then treated with or without TGF-β1 and/or TNF-α for 18 h. The cells were harvested, and the luciferase activity assay was performed. Data are mean ± SD. *P < 0.05 *vs.* control; ^#^ *P* < 0.05 *vs.* TGF-β1 0.2 ng/ml + TNF-α 0.2 ng/ml; ^$^ *P* < 0.05 *vs.* TNF-α 0.2 ng/ml + TGF-β1 0.2 ng/ml + SP600125 or TNF-α 0.2 ng/ml + TGF-β1 0.2 ng/ml + SIS3. Experiments were repeated three times.

To further investigate the mechanism(s) how Smad3 and JNK1/2 signaling modulates the expression of PGC-1α, we employed PGC-1α promoter luciferase assay. Both TGF-β1 and TNF-α decreased PGC-1α promoter activity in a dose-dependent fashion in NRK52E cells (**Figure 10A**). TGF-β1 and TNF-α had additive effects on PGC-1α promoter activity and SIS3, and Sp600125 can reduce the inhibitive effects TGF-β1 and TNF-α on PGC-1α promoter activity in NRK52E cells (**Figure 10B**). TGF-β1 increased PGC-1α promoter activity in a dose-dependent fashion in NRK49F cells. Low dose of TNF-α increased while high dose of TNF-α decreased PGC-1α promoter activity in NRK49F cells (**Figures 10C, D**). Treatment with low dose of TGF-β1 and TNF-α had an additive effects on PGC-1α promoter activity while SIS3 and SP600125 blocked the effects of TGF-β1 and TNF-α on PGC-1α promoter activity in NRK49F cells (**Figures 10C, D**).

## Discussion

Tubulointerstitial damage is the common pathway to ESRD while inflammation and fibrogenesis are the two major determinants of the progression from CKD to ESRD (Li et al., 2006; Picken et al., 2016). The roles of JNK and Smad3 signaling pathways in inflammation and fibrogenesis have been studied on an individual basis (Sato et al., 2003; De Borst et al., 2007; Ma et al., 2007; Li et al., 2010). In the present study, we demonstrated that activated JNK and Smad3 signaling pathways were co-localized in the tubulointerstitial compartment after FA-induced kidney damage, which provided rationale that blockade of both Smad3 and JNK signaling pathways may provide better protection from FA-induced progressive CKD than single blockade of Smad3 signaling or JNK signaling. Co-administration of SP600125 and SIS3 was highly effective in blocking each signaling pathways and protected tubular epithelial cells from apoptosis and promoted tubular epithelial cell but decreased renal fibroblast proliferation through modulation expression of PGC-1α in both tubular epithelial cells and renal fibroblasts at transcriptional level. Co-administration of SP600125 and SIS3 achieved better renal function protection from FA-induced progressive fibrosis and inflammation than that of single blockade.

Tubular atrophy is an important characteristic of progressive CKD. In the present study, we demonstrated dynamic changes of mitochondrial cytochrome c, which reflects mitochondrial function, and cytosol cytochrome c, which indicates mitochondrial injury after FA-induced kidney damage. We further demonstrated that blockade of Smad3 or/and JNK signaling increased mitochondrial cytochrome c and decreased the interaction between cytochrome c and Apaf-1. The interaction between cytosol cytochrome c and Apaf-1 is the initial step in the cytochrome c/Apaf-1/procaspase-9/procaspase-3 activation sequence in the apoptosis pathway (Li et al., 1997; Zou et al., 1997). Tournier et al. demonstrated that JNK signaling activation is required for stress-induced activation of the cytochrome c-mediated apoptosis in primary murine embryonic fibroblasts (Tournier et al., 2000) while Smad3/Nox4-mediated mitochondrial dysfunction plays a critical role in puromycin-induced podocyte injury (Yu et al., 2014). In our study, blockade of Smad3 and JNK signaling pathways further decreased the production of cytosol cytochrome c, the interaction between Apaf-1 and cytochrome c and active caspase-3 production, which accounted for the reduction in tubular epithelial cell apoptosis in FA-induced CKD. Thus, blockade of JNK and Smad3 signaling may rescue tubular epithelial cells from apoptosis.

Fully functional mitochondria is essential to the preservation of normal renal function (Tabara et al., 2014; Ralto and Parikh, 2016). PGC-1α plays an essential role in controlling MB and function (Wu et al., 1999). Downregulation of PGC-1α mRNA or/and protein levels was observed in several models of early stage AKI, including FA-induced, cisplatin-induced, and ischemia-reperfusion-induced kidney injury (Funk and Schnellmann, 2013; Ruiz-Andres et al., 2016). PGC-1α-deficient mice suffered persistent injury following endotoxemia while overexpression of PGC-1α after oxidative stress accelerated recovery of tubular cells (Rasbach and Schnellmann, 2007; Tran et al., 2011). Thus, the insufficiency of PGC-1α in tubular cells may contribute to persistence of injury and tubular atrophy. However, the upregulation of PGC-1α was also observed in UUO (Hickey et al., 2011) and recovery phase of ischemia––reperfusion-induced kidney injury (Funk and Schnellmann, 2012; Funk and Schnellmann, 2013). Our *in vivo* studies clearly demonstrated that the expression of PGC-1α was decreased in tubular epithelial cells while increased in myofibroblasts in FA-induced progressive tubulointerstitial damage. This up- *versus* down-regulation of PGC-1α in the two cell types reflects the pathologic changes during tubulointerstitial damage, tubular epithelial cell atrophy, and fibroblast proliferation. Our *in vitro* studies further showed that knockdown of PGC-1α reduced EGF-induced epithelial cell proliferation and PDGF-BB-induced fibroblast proliferation. Thus, our study provided evidence that the expression level of PGC-1α was correlated with proliferation of both tubular epithelial cells and renal fibroblasts. The tempo-spatial changes of PGC-1α expression are correlated with pathological changes in progressive CKD: tubular atrophy, and myofibroblast proliferation and accumulation. Co-administration of SP600125 and SIS3 prevented the up-regulation of PGC-1α expression in myofibroblasts and down-regulation of PGC-1α expression in tubular epithelial cells in FA-induced progressive tubulointerstitial injury. *In vitro* studies further demonstrated that both TGF-β1 and TNF-α downregulated PGC-1α expression in tubular epithelial cells while upregulated PGC-1α expression in renal fibroblasts. JNK inhibitor SP600125 and Smad3 inhibitor SIS3 abrogated the impact of TGF-β1 and TNF-α on the expression of PGC-1α in tubular epithelial cells and renal fibroblasts, which accounted for increased tubular proliferation with better repair and less fibroblast proliferation with the reduced accumulation of myofibroblasts and less fibrosis. Taken together, our study demonstrated a whole picture of tempo-spatial expression of PGC-1α in progressive CKD model and the exact role of PGC-1 in tubular epithelial cells and renal fibroblasts. Our studies also suggested that blockade of JNK and Smad3 signaling may promote tubular epithelial proliferation but inhibit fibroblast proliferation through modulation expression of PGC-1α.

PGC-1α is a transcriptional coactivator that regulates genes involved in MB and energy metabolism and provides a direct link between external stimuli and the modulation of mitochondrial function (Lin et al., 2005). Smad3 directly binds to Smad-binding elements (SBEs) on gene promoters to regulate target gene transcription (Feng and Derynck, 2005) while activation of JNK signaling leads to phosphorylated c-Jun binding to AP-1 binding site (Chung et al., 1996). Sequence analysis revealed the presence of SBE and AP-1 binding sequence and other transcription factor binding sequence on the PGC-1α promoter (Handschin et al., 2003). In the present study, we demonstrated that TGF-β1 or TNF-α modulated PGC-1α promoter activity in a cellular type-dependent fashion, suggesting that other factors in the transcription complex are involved which either enhance or block transcription. Blockade of both Smad3 and JNK signaling pathways increased or decreased PGC-1α transcriptional activities in tubular epithelial cells or fibroblasts, respectively, further supported the notion that blockade of both JNK and Smad3 signaling pathways provides better protection from progressive tubulointerstitial damage than single blockade.

In conclusion, our studies demonstrated that blockade of both JNK and Smad3 signalings achieved better protection from FA-induced progressive renal tubulointerstitial damage than single blockade. Inhibition of Smad3 and JNK signaling decreased cytosol cytochrome c production and apoptosis, modulated PGC-1α expression and mitochondrial metabolism, promoted tubular recovery, and reduced fibroblast accumulation. Our study suggested that blockade of both JNK and Smad3 signaling pathways may be a novel therapeutic strategy for progressive CKD.

## Data Availability

All datasets generated for this study are included in the manuscript.

## Ethics Statement

This study was carried out in accordance with the recommendations of the Human Ethics Committee of Monash Medical Centre and the Human Ethics Committee of the 1st Affiliated Hospital, Sun Yat-sen University with written informed consent from all subjects. All subjects gave written informed consent in accordance with the Declaration of Helsinki. The protocol was approved by the Human Ethics Committee of Monash Medical Centre and the Human Ethics Committee of the 1st Affiliated Hospital, Sun Yat-sen University. This animal study was carried out in accordance with the recommendations of the Sun Yat-sen University Animal Ethics Committee. The protocol was approved by the the Sun Yat-sen University Animal Ethics Committee.

## Author Contributions

Conceived and designed the experiments: JL. Performed the experiments: MJ, JF, XQ, SL, YS, YC, JL. Analyzed the data: MJ, JF, XQ, SL, SN, YS, YC, DY, DL, B-CL, MT, WC, YR, DN-P, XJ, JL, XY. Contributed reagents/materials/analysis tools: JL. Wrote the paper: JL, DN-P.

## Funding

This study was supported by the National Natural Scientific Foundation of China (NSFC No. 81670667 to JL, 81670648 to XJ, 81800605 to MJ and 81600513 to DL), Guangdong Medical University of Provincial and Municipal Construction of Colleges and Universities Project (NO. 4SG18001Ga to JL) and the National Health and Medical Research Council (NHMRC) of Australia (APP1057581 to JL). MJ was supported by the International Program for Ph.D. Candidates, Sun Yat-sen University. DN-P has previously received funding from Celgene for studies of JNK inhibitors. The funder played no part in this study.

## Conflict of Interest Statement

The authors declare that the research was conducted in the absence of any commercial or financial relationships that could be construed as a potential conflict of interest.
